# Supplementary material for: A high-resolution mRNA expression time course of embryonic development in zebrafish
Source: eLife. 2017 Nov 16;6:e30860. doi: 10.7554/eLife.30860 (PMC5690287; doi:10.7554/eLife.30860)
Supplement: Supplementary file 6. [file elife-30860-supp6.zip › biolayout-clusters-files/Cluster152-genes.html]

Cluster152


# Cluster152: Genes

| Ensembl ID | Gene Name | Chr | Start | End | Biotype |
| --- | --- | --- | --- | --- | --- |
| ENSDARG00000046107 | CASKIN1 (1 of many) | 3 | 35503265 | 35671638 | protein\_coding |
| ENSDARG00000088124 | adrb2a | 14 | 34485151 | 34490301 | protein\_coding |
| ENSDARG00000103333 | baiap2b | 12 | 35911987 | 35998262 | protein\_coding |
| ENSDARG00000061051 | brinp3a.2 | 6 | 36152230 | 36219995 | protein\_coding |
| ENSDARG00000058585 | chst2b | 24 | 5754935 | 5757430 | protein\_coding |
| ENSDARG00000012671 | inhbaa | 24 | 3191762 | 3204082 | protein\_coding |
| ENSDARG00000001953 | pfkfb3 | 4 | 25483768 | 25496261 | protein\_coding |
| ENSDARG00000030547 | rnd1 | 23 | 17874456 | 17888435 | protein\_coding |
| ENSDARG00000035887 | zgc:91944 | 19 | 32984683 | 33013842 | protein\_coding |
